# The Use of Thermal Techniques in the Characterization of Bio-Sourced Polymers

**DOI:** 10.3390/ma14071686

**Published:** 2021-03-30

**Authors:** Ignazio Blanco, Valentina Siracusa

**Affiliations:** 1 Department of Civil Engineering and Architecture, University of Catania, Viale Andrea Doria 6, 95125 Catania, Italy; 2 UdR-Catania-National Interuniversity Consortium of Materials Science and Technology (INSTM), Viale Andrea Doria 6, 95125 Catania, Italy; 3 Department of Chemical Sciences, University of Catania, Viale Andrea Doria 6, 95125 Catania, Italy; vsiracus@dmfci.unict.it

**Keywords:** bio-polymers, thermal behaviour, recycling, thermal properties, sustainability

## Abstract

The public pressure about the problems derived from the environmental issues increasingly pushes the research areas, of both industrial and academic sectors, to design material architectures with more and more foundations and reinforcements derived from renewable sources. In these efforts, researchers make extensive and profound use of thermal analysis. Among the different techniques available, thermal analysis offers, in addition to high accuracy in the measurement, smartness of execution, allowing to obtain with a very limited quantity of material precious information regarding the property–structure correlation, essential not only in the production process, but overall, in the design one. Thus, techniques such as differential scanning calorimetry (DSC), differential thermal analysis (DTA), dynamic mechanical analysis (DMA) and thermogravimetric analysis (TGA) were, are, and will be used in this transition from fossil feedstock to renewable ones, and in the development on new manufacturing processes such as those of additive manufacturing (AM). In this review, we report the state of the art of the last two years, as regards the use of thermal techniques in biopolymer design, polymer recycling, and the preparation of recyclable polymers as well as potential tools for biopolymer design in AM. For each study, we highlight how the most known thermal parameters, namely glass transition temperature (*T*_g_), melting temperature (*T*_f_), crystallization temperature (*T*_c_) and percentage (%c), initial decomposition temperature (*T*_i_), temperature at maximum mass loss rate (*T*_m_), and tan *δ*, helped the researchers in understanding the characteristics of the investigated materials and the right way to the best design and preparation.

## 1. Introduction

A lack in the correct design of polymeric materials made since the 1970s, without foreseeing their end of life and indeed making efforts to extend their shelf-life as long as possible, is at the basis of the environmental problems connected to the disposal of plastic waste we have today [1,2]. Starting from this consideration, the world population is torn between the desire to save the environment and the need to have functional products, at an affordable cost, which only plastic materials can provide. This inner debate is, in most cases, influenced by erroneous beliefs, such as: only bio-derived plastic can be considered biodegradable or degradable in a short time; the cost associated with fossil-derived plastic is much lower than that of biopolymers. As regards this latter, the history of plastic testifies the absolute non-truthfulness. The early last century saw the affirmation on the market of Cellophane™ and Viscose™ products, obtained by processing natural substances, which successfully resisted the take-off of the petrochemical industry, owing to their competitiveness as regards the technological properties and economic viability. Thus, if we look back, the urban legends around the expensiveness of renewable polymers dissolve in the face of the evidence that the adoption of bio-based plastics in the market must be motivated by their functional properties and not merely by their green credentials [3]. The biodegradability and/or the ability of the environment to take charge of the exhausted polymer is strictly linked to the modifications made to it, in order to tune their properties as a function of the use for which it was designed. It is therefore not the nature of the material that determines its persistence in the environment, but the modifications it has undergone, such as surface modification, additives, coating etc. Thus, following these considerations, the interest of companies to move towards biopolymers is driven more by the awareness of having to replace a depleted reservoir like crude oil than by a green-consciousness. Anyway, beyond the reasons for which it was made, it is still a choice of sustainability and the development of a platform of molecules from renewable resources, able to lead to biopolymers, is laying the foundation for a sustainable future [4,5]. This new challenge is very clear: each management decision (industrial, academic, political) must be integrated and supported by environmental considerations, thus the need to gain any possible information about the material’s impact on the environment. Among the different tools to evaluate the sustainability of a material, life cycle assessment (LCA) emerges in the last decade over the other ones, providing the assessment of the environmental impacts and monitoring the resources used during the production cycle of the goods, starting from the raw material up to the waste management [6]. Thus, before design, production and commercialization of biopolymers LCA studies are carried out to certificate their sustainability [7,8]. Probably the hot spot of this chain, staring from design and finishing to commercialization, is represented by the quality control methods in the development and manufacture of polymeric materials. To this aim, thermal analysis (TA) is recognized as one of the most useful investigation tools, for measuring the physical properties of the material, for clarifying its thermal and mechanical history, for characterizing and designing processes used in its manufacture, and for estimating its lifetimes. From a practical point of view, measuring the material response when heated or cooled is the connecting link of the various techniques constituting TA, reported in Table 1 as a function of the physical property measured [9].

This review would offer an overview of the application of TA techniques in the design and characterization of biopolymers in the emerging area of the materials science.

## 2. Thermal Techniques in Biopolymers’ Design

The use of renewable materials to design biopolymers have attracted increasing attention in recent years due to the environmental consciousness of people. This urgent need in developing new green design is related to the huge amounts of plastics obtained from fossil sources ending up as wastes producing large amounts of carbon dioxide and contributing to global warming or accumulating in the environment where do not degrade, or degrade slowly, thus altering the biome. Furthermore, biopolymers offer a possible alternative to the classic polymers, especially in short life-time application and when their recycling is difficult and/or not economical.

Schlemmer and Araujo Sales aiming at produce materials with low environmental impact, designed thermoplastic starch (TPS) by the means of casting process using cassava starch and a plasticizer derived by vegetable oils of Brazilian Cerrado, namely Buriti (Mauritia flexuosa), macauba (Acrocomia aculeata), and pequi (Caryocar brasiliense) [10]. The prepared biopolymers were analyzed by TGA, DSC, and TMA. The thermomechanical characterization, they carried out, showed homogeneity and general good thermal behaviour strongly influenced by the type of oil used, since they observed that the increase in the concentration of unsaturated fatty acids led to a worse thermal stability. In this study, the TGA turned out a key technique because by analyzing the different mass loss stages the authors were able to set a suitable design. By comparing TGA profiles of starch and films they observed, for this latter, an increase in mass loss, due to the presence of oil in the matrix leading to a molecular re-organization, with less exposed hydroxyl groups. Proceeding with the analysis of the thermograms they observed how the weaker intermolecular starch–oil bonds caused an anticipation of the last decomposition step. In their thermal investigation, DSC scan presented a shoulder in the melting endotherms of original crystallites, attributed by the authors to a lamellar rearrangement during the crystallization process. In addition, TMA showed them a greater resistance to the penetration when the oil was added to the film, due to the reduction of the intermolecular interactions. This enhancement of the chains’ distance affected the mechanical properties of the films, in particular the resistance to the penetration. They supported the use of the Brazilian Cerrado’s oil, because of the relevant plasticizer effect for cassava starch highlighted by DSC and TMA, in the production of less environmentally impactful materials.

**Figure 1:**
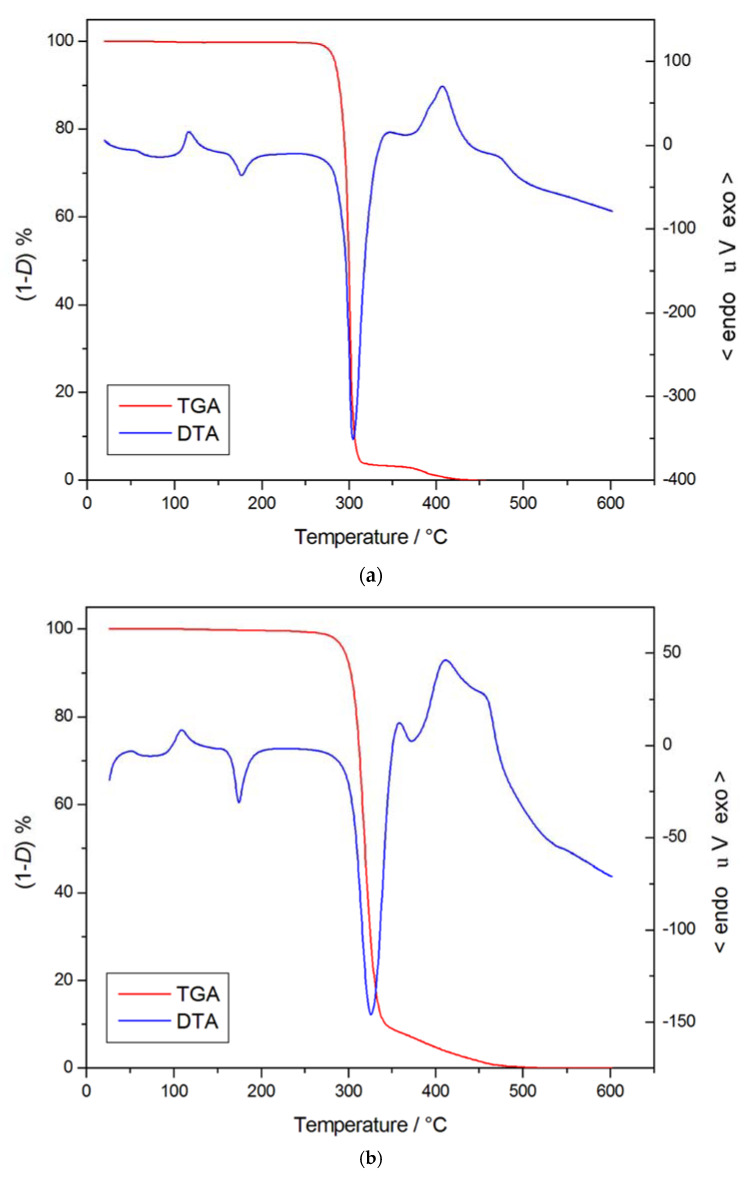

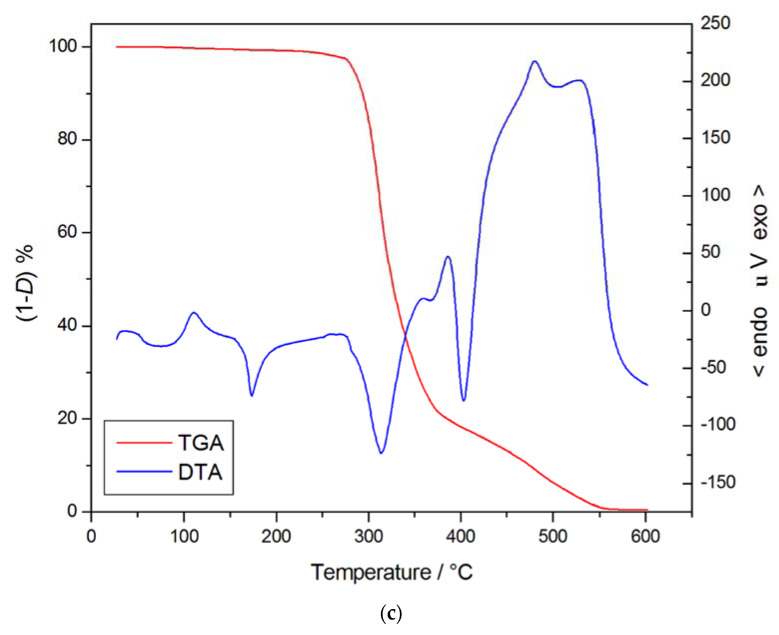
TG/DTA curves for Liquid Wood (**a**), PLA/lignin 10 mass% blend. (**b**) and PLA/lignin 25 mass% blend (**c**). Reprinted from [11] with permission of Springer Nature

Cicala et al. investigated, by using thermogravimetric/differential thermal analysis (TG/DTA), the effect of processing temperature and time on polylactide (PLA)/lignin blends. A completely bio-blend by mixing PLA with different lignin contents was designed [11], by processing at 190 and 170 °C for different mixing times. TGA allowed them to observe the influence of lignin content in the degradation of the prepared blends, in comparison with a commercial one containing PLA, soda lignin (5%), and softwood (15.4%). In particular, they observed for the blends containing lignin, differently than the commercial one with softwood, good processing stability attributing the improved stability and suggesting not only good processing condition but also promising recyclability of the PLA/lignin blends. The role of TGA for these Italian researchers was determining in defining the best PLA/lignin ratio, correlating the stability of the bend with the initial decomposition temperature (*T*_i_) and temperature at maximum mass loss rate (*T*_m_). They recorded an increase in both parameters when 10% of lignin was added to the matrix (Figure 1), whilst further addition of lignin up to 25% negatively affected the heat resistance and the degradation rate.

Fardim and co-workers used thermal techniques in the design and characterization of bio-based foam by using acrylated epoxidized soybean oil and cellulose fibres at different percentages [12]. In the preparation of their bio-based architecture, they created a three-dimensional polymer foam inserting the cellulose fibres, who acted simultaneously as reinforcing agent and foam stabilizer, since hindered the drainage of liquid from the film in the gas-oil interfaces. The constancy of *T*_i_ and the relatively low quantity of solid residue at 300 °C measured by TGA made the authors ascertain that the inclusion of cellulose and the unreacted components (responsible for the residue) did not affect the thermal behavior of the cured foams. Furthermore, the residue obtained at the end of TG scan allowed the authors to speculate about the degradation mechanism. They hypothesized the carbonisation of the fibres since the C content was higher for the residue of the samples containing cellulose with respect to that of the net soybean oil polymer.

With the final goal of producing cost-effective polymeric composites in order to address pollution issues and develop a product with superior properties Beber et al. projected to prepare biodegradable blended with low-price organic fillers. Polyhydroxybutyrate (PHB) and poly(butyleneadipate-co-terephatalate) (PBAT) were melted extruded with 20% of Babassu, an indigenous tree of some regions of Brazil. By using DSC technique, they observed the crystallization inhibition, whilst the use of Babassu, which did not affect the melting behaviour, allowed the tuning of crystallization and the plasticization [13]. They demonstrated the key role of DSC measurements in keeping the solid state at higher temperatures for designing products able to work at high temperatures. They observed for the blends a shift of PBAT melting peak toward higher temperatures with respect to pristine polymer, thus assuming that the heat transfer mechanisms were hampered in the blends. Since the macromolecules and crystallites of PHB and PBAT are in a mixed-interlaced-solid state, when PBAT started to melt PHB chains and crystals were still solid, thus hindering the heat transfer and free movement of polymer chains leading to an increase of energy needed to melt the PBAT/PHB blends.

**Figure 2:**
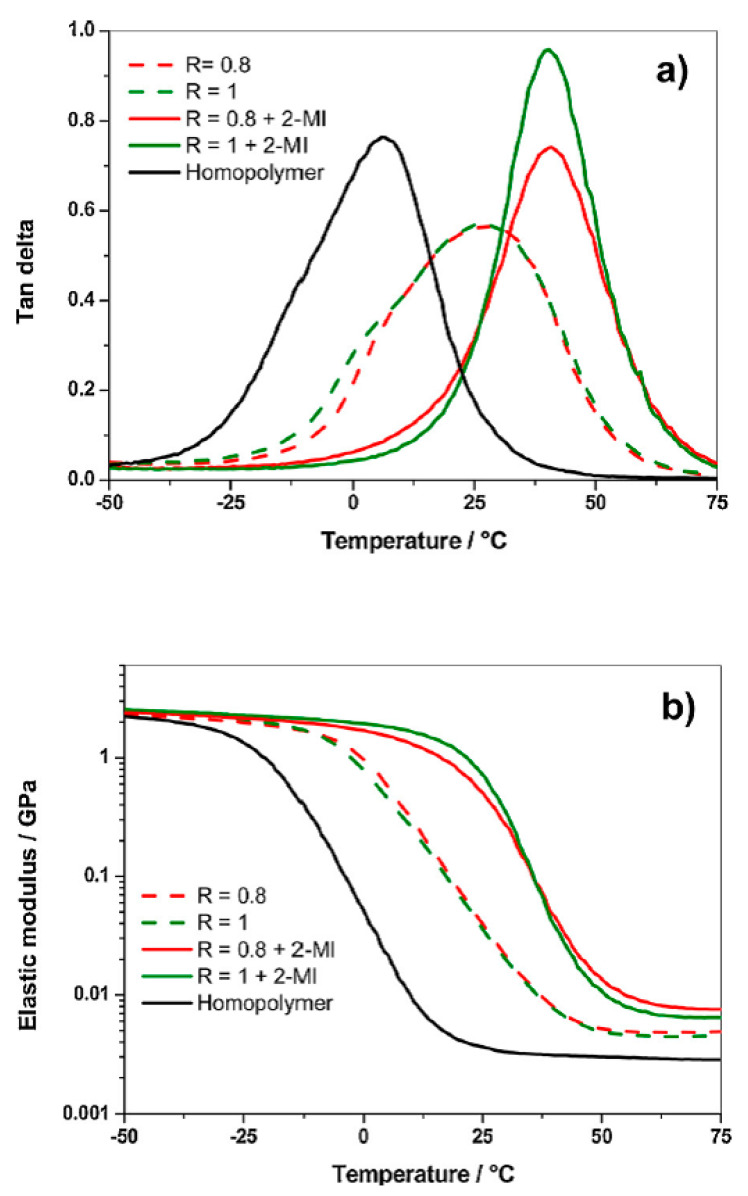
(**a**) tan delta curves and (**b**) elastic modulus vs. temperature from −50 °C to 75 °C of cured Epoxidized Linseed Oil (ELO)/bio-based dicarboxylic acid at different ratio (R) with 2-methylimidazole (2-MI). Reprinted from [4] with permission of Elsevier.

The importance of the DSC and dynamic mechanical analysis (DMA) tools has been reported by Mija and co-workers, who recently focused their studies on the utilization of bio-resources to design bio-based epoxy resins. They used dicarboxylic acid as curing agent and synthesized the hardener from maleic anhydride and dipropylene glycol and then copolymerized with epoxidized linseed oil (ELO) [4]. A deep investigation of the copolymerization reaction was carried out by DSC and DMA, showing the influence of the epoxy/dicarboxylic acid ratio and highlighting the selective action of the imidazole derivative in the enhancement of the crosslink density. The a-relaxation was the factor, investigated by means of DMA, allowing the authors to design the best thermosets formulation. In particular, the α-relaxation, correctly linked by the authors to the cooperative motions of the main chains, was assimilated to the glass transition, thus giving direct indications on the viscoelastic state at room temperature, on mechanical properties, and the domain of applications of materials (Figure 2).

They also performed thermogravimetric measurements to show the excellent thermal resistance of the designed *ecoxy*. TGA scans confirmed how the different chemical structures affect the thermal performance, with a preference for ether bonds rather than the ester ones. Thus they were able to regulate the copolymer ratio by analyzing the difference in the starting of degradation, influenced by the high or less presence of ester bonds.

Thermal analysis on an innovative thin self-sustainable blend films made of polyaniline (PANI) and cashew gum (CG), an exudate polysaccharide largely present in Brazilian region, was carried out by da Cunha and his colleague with TGA and DSC. The thermal analysis on the biopolymers, prepared by casting method, revealed a positive affection of the thermal stability, because of the presence of CG, showing how the thermal behavior of PANI and CG was preserved in the blends, and pointing to the possibility of using this blend as an innovative conductive polymer [14]. In addition, by coupling the TGA mass loss with the thermal phenomena observed in the DSC profiles, they were able to make association with the volatilization of the adsorbed water molecules, with the evaporation of the solvent used in the film preparation, as well as with the polymer chain cross-links.

The kinetics of cashew gum degradation was evaluated by Gonçalves Mothé and Souza de Freitas. The Brazilian researcher estimated the activation energy and pre-exponential factor with well-known kinetics literature methods, giving a probable mechanism of decomposition and estimating the shell-life of the polysaccharide [15]. Also in this case, by combining TGA and DSC, the authors attributed the single degradation stages to a specific phenomenon and estimated the cashew gum lifetime in 20 years, by considering the temperature range between 40 and 80 °C.

**Figure 3:**
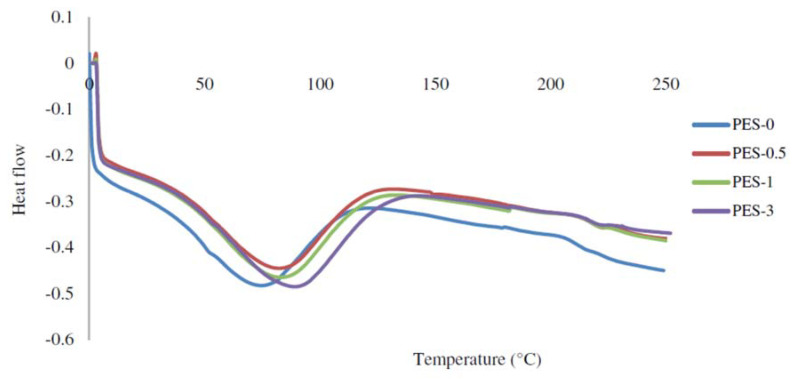
DSC thermograms of PES membranes embedded with different modified microcrystalline. Cellulose (MMCC) contents. Reprinted from [16] with permission of Elsevier.

Rafieian et al. carried out a thermal characterization on adsorptive membranes based on polyethersulfone (PES) filled with modified microcrystalline cellulose (MMCC) with the purpose to improve the membrane affinity for heavy metals during filtration of aqueous solutions. In this study TGA allowed to the authors not only to certified a thermal behavior sufficient to meet the performance requirements but also to work appropriately in the membrane design, being possible identify the main phase of membranes degradation. The authors reached another important achievement in membrane’s characterization, since DSC analysis showed an enhancement in the glass transition temperature (*T*_g_) (Figure 3) that they attributed to the reduction in polymer chains’ mobility for the increase in membrane interfacial interactions [16].

de Abreu Martins and her colleagues studied the possibility to add glycerol, derived from the transesterification process in biodiesel production, in epoxy adhesives. By using DMA they wanted to investigate if glycerol acted as plasticizing or not, when it was added to the epoxy at different percentages (1.0, 2.5, 5.0 and 10.0%). By adding plasticizer is expected a degree of freedom for the lateral groups of the polymer, with rotational movements occurring, because plasticizer, installed among the chains lead to an increase of the polymer free volume of the polymer. For this reason, the authors would have expected a reduction in the glass transition temperature (*T*_g_), but surprisingly they observed a gradual, although small, increase in *T*_g_ as a function of increasing glycerol percentages thus not recommending high percentage of use [17].

The research group in Palermo, led by Proff. Lazzara and Milioto, since some years are employed in the design and thermal characterization of clay reinforced biopolymers to be applied in different heterogeneous areas, from biomedicine to the restoration of cultural heritages. Aiming at introducing smart response abilities to the biomaterials, they proposed the incorporation of eco-friendly filler such as Halloysite nanotubes. By using TGA in a classical way evaluated the physicochemical properties finding improved mechanical and barrier properties, and good thermal behavior for the polysaccharides/halloysite nanocomposites but, above all, they investigated the thermodynamics of polymer/nanoparticle adsorption by nanocalorimetry. Since the reduction of configuration freedom of the polymer adsorbed onto the nanotubes led to a negative entropic contribution, the authors attributed the observed stabilization effect to the steric effect and strong binding energy [18].

**Figure 4:**
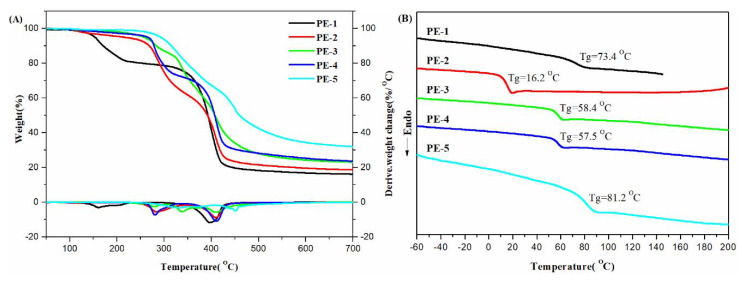
TGA curves (**A**) and differential scanning calorimetry (DSC) traces (**B**) of PEs. Reprinted from [19] with permission of MDPI.

**Figure 5:**
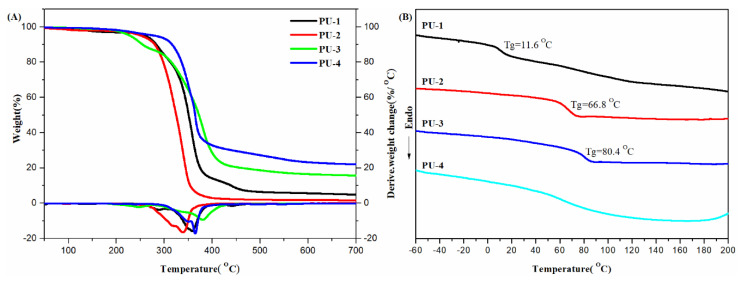
TGA curves (**A**) and differential scanning calorimetry (DSC) traces (**B**) of PUs. Reprinted from [19] with permission of MDPI.

A sustainable polymer chemistry was carried out by Xie and co-workers designing polymers by using biobased chemicals, through the direct reduction of vanillin, such as 4-(hydroxymethyl)-2-methoxyphenol and 2-(4-(hydroxymethyl)-2-methoxyphenoxy)ethanol. Then, after polycondensation and polyaddition, they prepared polyesters and polyurethanes and carried out a wide TGA and DSC characterization [19]. By looking at the *T*_g_ increasing (Figure 4 and Figure 5) the authors were able to set the better polymer formulation, since DSC measurements allowed them to correlated these results with the degree of flexibility (or rigidity) present in the polymer chain. The comparative analysis showed that the higher reactivity of the aliphatic hydroxyl group and the flexibility of the alkyl segment had a great influence on the thermal properties of polymer, as evidenced also by TGA. Once again, these authors demonstrated how the thermal techniques had proved to be an important tool for the design of new materials.

Rodriguez Saiz et al. carried out a wide thermal characterization on an eco-panel obtained by using industrial sub-products and the waste from Polyurethane Foam (PF) panels. By using High-Temperature Differential Scanning Calorimetry (HT-DSC), they measured the specific heat (*C*p) of the mortar with foams and studied the energy behavior of the new panels in service, i.e., used in a building. Since the material’s ability in storing energy depends not only by its mass and density but also by its specific heat, the HT-DSC allowed them to evaluate the material’s thermal inertia. Considering that the higher the thermal inertia, the greater the time for a polymer to reach thermal equilibrium with the surrounding media, they found good thermo-mechanical properties for the recycled materials [20].

**Figure 6:**
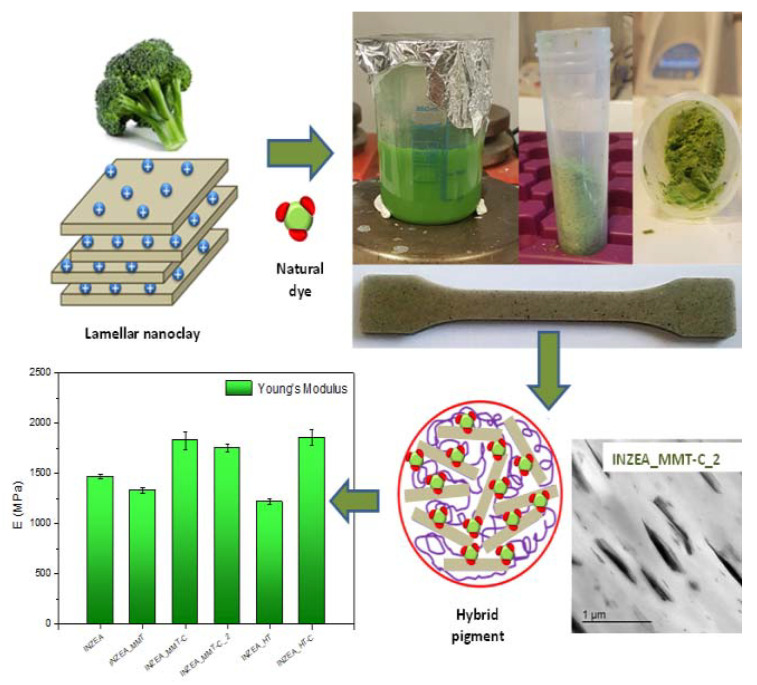
Hybrid nano-pigments extracted from broccoli waste. Reprinted from [21] with permission of MDPI.

Garrigos et al. investigated the chlorophyll extracted from broccoli waste to obtain hybrid nano-pigments for a possible bio-replacement of synthetic dyes (Figure 6).

TGA, DSC and dynamic mechanical thermal analysis (DMTA) allowed to these researchers to record a thermal stability increase for nanoclays filled with natural dyes to protect chlorophyll degradation [21]. Their TGA analysis also confirmed for the investigated biopolymer composites a partially intercalated/exfoliated structure, thus showing the potential of broccoli wastes as component for nanoclays bio-composites.

Lee and co-workers designed natural fibre-reinforced polymers for packaging and by the means of TGA and DSC they studied the effect of the bleaching treatment on the thermal performance of jackfruit fibre reinforced PLA composite. First of all, DSC allowed them to verify, by measuring melting, that the poor interfacial bonding of the fibre/matrix absorbs less heat energy before melting and this is found to be synchronised with the strength profile and lower mechanical properties. Always by using DSC they verified that the insertion of natural fibres reduced the *T*_g_ values as a function of increasing fibres content. Thus, aiming to increase the polymer wetting on the fibre surface, they performed bleaching treatments for removing non-cellulosic components on fibres surface. To verify the success of this treatment, DSC was again determinative because the increase of the physical entanglement resulted in higher *T*_g_ values. Moreover, by evaluating the characteristic parameters of TGA analysis, such as *T*_i_ and *T*_m_, they found a confirm about the success of bleaching treatment since the non-cellulosic components were removed from the fibre’s surface, thus improving their thermal performance and guarantying a longer service period [22].

Mothe et al. designed a new composite by using coffee capsule residue as a polymeric matrix reinforced with sugarcane bagasse fibers and evaluating thermal and mechanical behavior by the menas of TGA, DTA and DMA. They found a clear influence of the fiber content on the composite’s thermal behavior as well as on the viscoelastic properties. Also, in this case, the thermal technique helped the Brasilian researchers in associating the reduction in thermal stability with a higher presence of fibers in the composites, due to the increase in the hydrophilic components and thus the moisture absorption. DSC was also used to highlight the adhesion between fiber and matrix in the composites [23].

## 3. Thermal Techniques in Polymers’ Recycling and Preparation of Recyclable Polymers

Well know advantages, such as cheapness and ease of processability allowed in the last 30 years the suppression of the traditional materials in favor of the polymeric ones. Especially in the seventy and eighty, they were produced without considering their end of life, thus their recycling still is a current challenge including technological and economic issues [24]. Since the determination of chemical and physical properties of the materials used in a process represents the core of the good practices to meet the specifications required in the quality control and in order to have a satisfactory product from a sustainable view-point, the thermal techniques are widely employed in the design for recycling. Furthermore, the processing of recycled plastics cannot proceed without the help of these techniques, considering that before recycling a mixed waste stream, a procedure should be developed to “characterize” in a sound and scientific way, the polymeric components in the waste stream [25].

A pioneering work has been carried out by Rostek and Biernat describing the problems encountered in re-using waste plastics and tire rubber waste as raw materials for the preparation of liquid energy carriers for transport by using a derivatograph, an instrument that make the history of thermal Analysis in the eastern Europe. They focused their efforts in defining the kinetics of recycling processes, considering the heat necessary to break the polymer’s bonds and to create the new ones of the recycled material. The work of the two researchers was very important not so much for the results achieved as for the fact that they established, at the beginning of the recycling era, that was not fully possible to define the kinetics at each transformation stage, because of the complexity of plastic mixture waste. Their studies were carried out in order to pre-determine the kinetics of thermal decomposition, showing the complexity of the reactions occurring during decomposition and the enthalpy change of the process [26].

More recently, Vecchio Ciprioti and co-workers deeply investigated, by the means of thermal techniques, the plastic waste stream derived from electric and electronic equipment (WEEE) aiming at their correct disposal. They found a representative fraction of three polymers (acrylonitrile-butadiene-styrene; high impact polystyrene and polybutadiene terephthalate) and thus, they replicated this ternary polymer mixture and provided a Kinetics study of its degradation using TGA. Literature methods, namely the Kissinger and Ozawa–Flynn–Wall ones, were used to evaluate the thermogravimetric data, allowing to calculate the reaction time values to achieve the maximum pyrolysis rate in the three main components and in the real WEEE samples [27]. By means of calorimetry, they determined bot low (LHV) and high (HHV) heating value, and by taking into account humidity values their pyrolysis studies on WEEE demonstrated the possibility to convert the whole hydrocarbon content contained in the solid fraction into a fluid shape, whether liquid or gas. Furthermore, TGA analysis allowed them to exactly assess the ash content coming from both housing devices and virgin polymers. Continuing their studies, the Italian researchers investigated four synthetic mixtures thereof with different compositions representing commingled postconsumer plastic waste and WEEE waste using TG/DTA coupled with Fourier transform infrared spectroscopy (FTIR). They determined an overall energy (degradation heat) for the mixtures of about 4–5% of the exploitable energy of the input material. In addition, analyzing the TG’s evolved gas they assessed the reaction products as monomers or fragments of the polymeric chain [28].

Khaobang and Areeprasert used TGA to study kinetics of decomposition of High-Impact Polystyrene aiming at obtaining oil. Evaluating a series of thermogravimetric scans, they set the optimum pyrolysis conditions and the yield of oil recovery, finding that the higher pyrolysis temperature reduced viscosity and facilitated the thermal decomposition. Furthermore, the analysis of the pyrolysis products, by gas chromatography-mass spectrometry (GC-MS) analysis, allowed them the classification of the different stages of the process and, depending by the pyrolysis conditions, the oil composition. They also found that the activation energy of the produced oil was not affected by the pyrolysis temperature [29].

Evaluation of TGA data with kinetics models were carried out also by Santos et al. aiming at identifying the similarities and differences of paper and low-density polyethylene waste, since the recycled materials must have the same properties [30]. Due to the similarity of the waste’s materials studied, pre-exponential and activation energy were all in the same order of magnitude, and the reaction order showed a divergence that can be caused by the difference in the material structure. Hence, the optimization using a stochastic strategy was satisfactory with parameters close to the ones found by using the Kissinger and Ozawa method [31,32,33].

**Figure 7:**
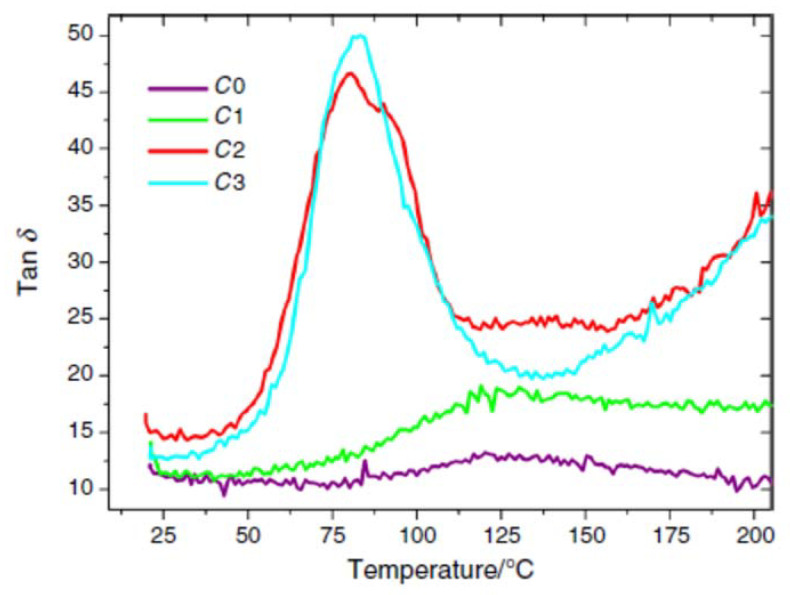
DMA curves of (C0) geopolymer with pure metakaolin; (C1) geopolymer with metakaolin/fly ash at 20%; (C2) geopolymer with metakaolin/fly ash at 50%; (C3) geopolymer with metakaolin/fly ash at 70%. Reprinted from [34] with permission of Springer Nature.

Catauro et al. employed thermal techniques, namely DMA, DSC and TGA, to evaluate the thermo-mechanical performance of a series of geopolymers prepared by using pure metakaolin and fly ash recycled. They observed a shift of the tan *δ* value towards high temperature, a behavior usually observed for relaxation phenomena like glass transition in polymers (Figure 7). Their feeling was confirmed by the decay recorded for the storage modulus (E’), the same behavior shown by polymers when relaxation phenomena like glass transition occur. By testing their recycled geopolymers by DSC, they certificated the irreversible nature of the endothermic transition, confirming the presence of relaxation phenomena at higher temperature [34].

In order to testify the reusability of a polyamide-6/Spandex-recycled poly (ethylene terephthalate) melt-extrusion blends, Mendes et al. used TGA, DSC, and DMA to verify the miscibility of this blend and thus its employment for other textile applications. They used DSC and DMA in verifying the miscibility of the components and if the working temperature was comparable to that of virgin material. They used parameters such as crystallization and melting temperatures, as well as the degree of crystallinity of the recycled polymers to set the best blend composition. Once they reached the optimum design, they used TGA to verify the resistance to the thermal degradation, by measuring *T*_i_ and *T*_g_ by DMA. This latter technique showed two intermediate glass transition temperatures, resulting in blends with at least two phases confirming the miscibility [35].

A wide employment of the thermal techniques in the design of bio-based epoxies has been done by the research group at the University of Nice led by Prof. Mija. They designed new epoxidized vegetable oils (EVOs) that reacted with a disulfide-based aromatic dicarboxylic acid (DCA) to produce thermoset materials with recyclability properties. DSC, DMA and TGA were applied to investigate the structure−reactivity correlation and the thermomechanical properties of the prepared thermosets. They were able to determine, for the samples under investigation, homogeneous networks since a single tan *δ* peak was measured, as in the case of *T*_g_ by DSC. In particular, the parallel use of the two techniques (DSC and DMA) allowed the authors to observe a linear increase of the tan *δ* and *T*_g_ values as a function of the epoxy content in the copolymer’s formulation. By using TGA, they evaluated the thermal behavior of the different prepared epoxy systems. For the resins with low epoxy content they found the best results in term of resistance to the thermal degradation by considering the statistic heat-resistant index temperature (*T*_s_) and the temperature at 5% mass loss (*T*_5%_). Contrariwise, increasing the epoxy content, they observed a decrease in the thermal stability, explaining this behavior with the presence of the hardener (2,2′-dithiodibenzoic acid, DTBA) in the network, and consequently to the S−S bonds in the resins. The hypothesis of the authors is corrected if we consider that it is well known from the literature that the presence of S−S bonds thermally destabilizes thermosets because their lower dissociation energy compared with that of C−C bonds [36]. In addition, the French researchers correctly considered the effect of the oxirane ring content, that lead to the formation of more ester and hydroxyl groups during the curing, thus contributing to the thermal scissions of the networks [37]. Since they found a strictly link among EVO functionality, reactivity, cross-linking density, and final performances, they continued their studies by proposing the synthesis and thermal characterization of a reprocessable thermosets, whose recyclability was designed through a dual mechanism (i.e., disulfide metathesis; transesterifications), obtaining similar performance compared to the control ones. Also, in this case, they determined the protocol of the curing and post-curing processes for each formulation by using DSC. A cross-linking study, in fact, revealed the initiator efficiency by lowering the activation energy of the reaction and accelerating the curing rate [38].

Lejeail and Fischer used DSC in the design and thermal characterization of a completely recyclable epoxy-based thermoset composite materials involving the separation, recovery, and complete reuse of both components of the composite, the resin and the fibers [39]. Rheological and DSC studies, allowed them to verify the obtaining of a densely cross-linked polymeric networks and to observing the ability to uncross-link and thus regaining fluid behavior at elevated temperatures. In particular, by cyclic rheological studies and DSC, they assessed for their systems (and differently with respect to the permanent covalently cross-linked structure of a conventional epoxy system) the key points for the self-healing effect, i.e., the flow ability, the capability of restore cracks, and the resin fiber delamination sites at elevated temperatures. During the rheological analysis, they observed a decrease in the viscosity of the resin by increasing the temperature, with a temperature regime dominated by the r- Diels–Alder (DA) reaction during which the reversible cross-links are broken, and the material behaves as a polymer melt. DSC investigation was determining in verifying the occurrence of the r-DA reaction. They observed for the half-cross-linked resin two different transitions at about 100 °C (prominent) and at about 122 °C, corresponding to the r-DA reaction of the two stereoisomers, the endo- and the exo-form of the DA adduct.

## 4. Thermal Techniques in Polymers’ Bio-Reinforcement

If on one side is desirable a fast deterioration of the plastic to lighten the environmental burdens, on the other side many applications need high resistant polymers. Green polymers can be considered of great importance in terms of sustainable development, but they have shown low thermal performance with a fast thermal degradation process [40]. For this reason, many efforts were made by researchers in enhance their thermal resistance by adding bio-reinforcements.

**Figure 8:**
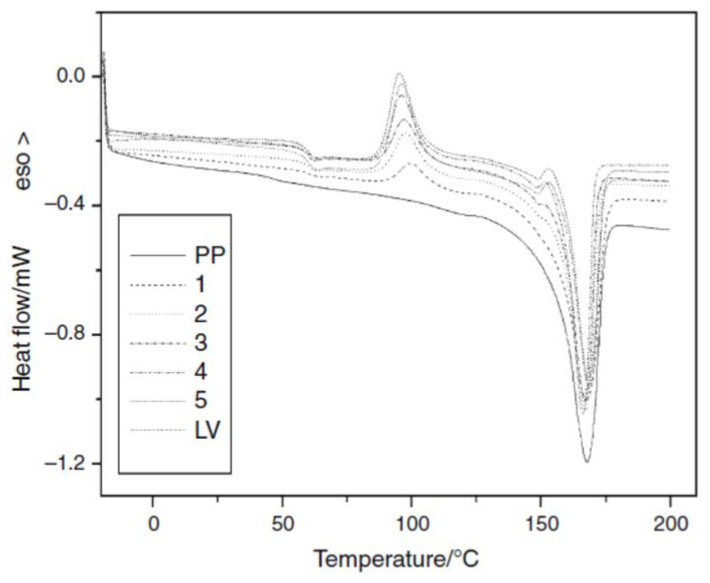
DSC curves, at 10 °C min^−1^, of pure polypropylene (PP), liquid wood (LV) and PP blends at 20% (1), 40% (2), 50% (3), 60% (4), 80% (5) of LV. Reprinted from [41] with permission of Springer Nature.

Due to its chemical stability, Polypropylene (PP) obtained the non-positive record of the increasing amount of plastic wastes on earth. For this reason, commercial Liquid Wood (a mixture of cellulose, hemp, fax and lignin) was used by Ziegmann et al. to prepare a more suistanable blend of PP. DSC and TGA were performed to study the influence of the blend’s composition on the thermal properties. DSC tools also in this case resulted a key tecnique for understanding the composition of the commercial formulation. Besides the melting, that shift towards higher temperatures on increasing the liquid wood (LV) content, they observed a *T*_g_ at about 60 °C and an exothermal peak at about 90 °C, becoming more evident with increasing the LV content in the blends. They attributed these phenomena to the presence in the commercial formulation of polylactic acid (PLA) (probably added to improve the workability of Liquid Wood), also supported by the DSC melting peak of virgin LV (Figure 8) that falls in the melting range of PLA.

Furthermore, TGA analysis allowed them to conclude that the use of polypropylene as matrix for their innovative blends not limited the LV’s processing temperature and did not anticipate the degradation of lignin incorporated in the blends, which may lead to a perfect marketable composite from these components [41]. Once established a thermal procedure protocol by DSC and TGA, the same research group verified the thermo-mechanical performance of the blend after a serious of recycling cycles. They observed a reduced intensity for the exothermic peak of the recycled sample as the recycling steps progressed, contrariwise they not observed significant differences for *T*_g_, they also verified the trend of DSC data by DMA, finding a good agreement. By performing TGA analysis and recording the same *T*_i_ for the virgin sample and the recycled one, they concluded that the recycling steps used had minor effects on the thermal stability of the recycled compounds [42].

Ge et al. prepared a completely biodegradable blend by reinforcing poly (lactic acid) (PLA) with lignin. DMA and TGA/DSC simultaneous analysis were carried out in order to verify the effects of lignin adding on the thermal behaviour of the blend. A coupled use of thermal techniques helped them to speculate about amorphous and crystalline phases in the blend. With DMA analysis they observed an increase in tan δ, which was significantly influenced by lignin, in the presence of initiator, due to the reduced crystallinity of PLA in the polymer blends. They proceeded in the characterization by performing TGA, demonstrating that the PLA degradation was not hindered by the presence of lignin, probably because the PLA-lignin interaction was not strong enough at the investigated temperatures. Downstream of the thermogravimetric experiments they speculated about the activation of PLA degradation by the abundant hydroxyl groups of the lignin, through thermohydrolysis and β-scission, which led to the lower molecular weight of PLA and the poorer thermal stability of the modified blends. In conclusion they observed a general increase of the mechanical strength but not an improvement in thermal stability and a lowering of glass transition temperature and melting temperature compared to pure PLA [43].

**Figure 9:**
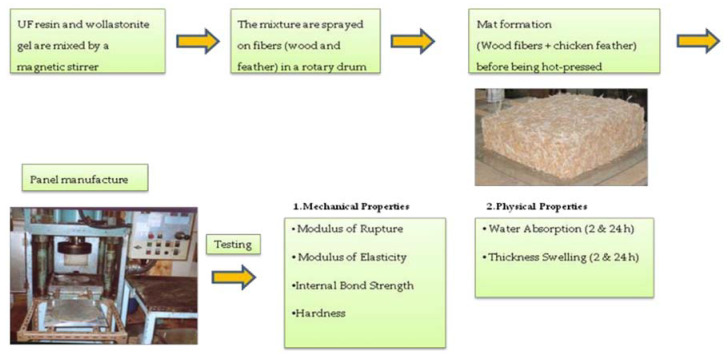
Flow diagram of the experimental procedure for the preparation of the wood panel reinforced with chicken feathers fibers. Reprinted from [44] with permission of MDPI.

The physical properties of a wood panel reinforced with sustainable fibers (Figure 9), such as chicken feathers, were investigated by Papadopoullus et al. [44].

**Figure 10:**
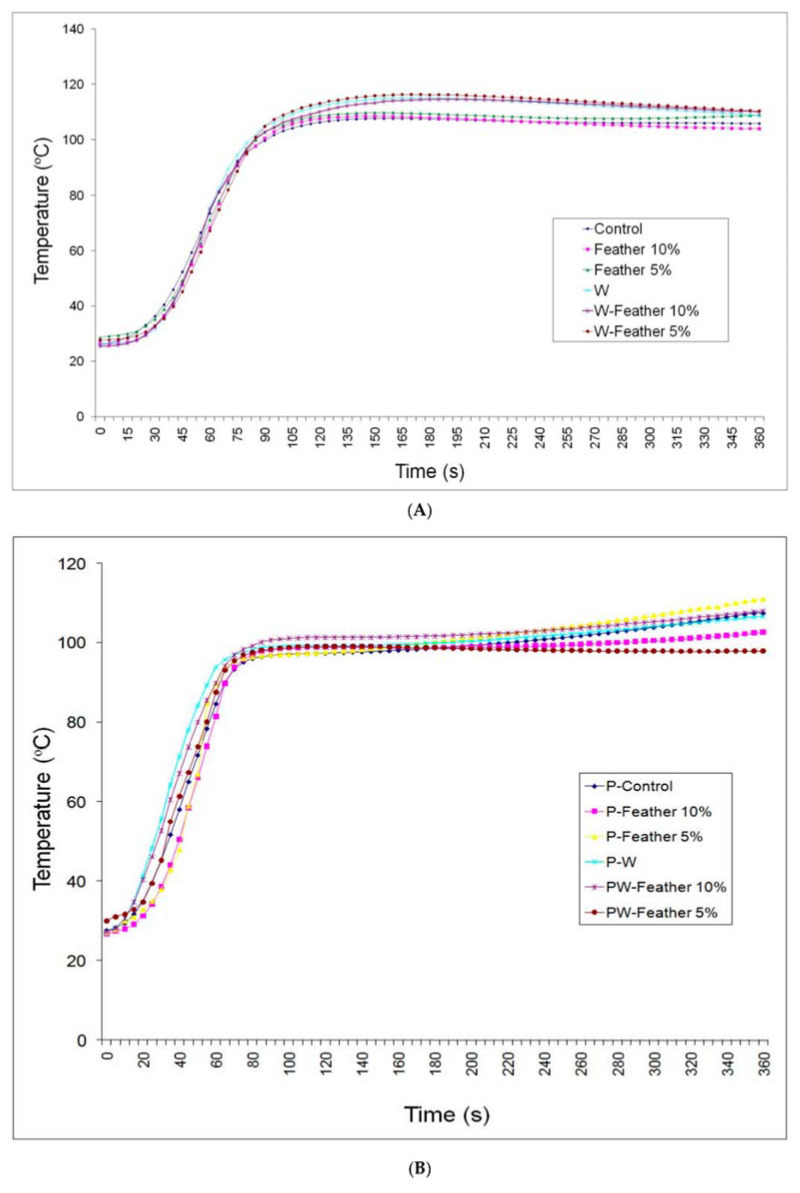
Temperature (Celsius) at the core section of the MDF (**A**), and the particleboard (**B**) at 5-s intervals (P = particleboard; MDF = medium-density fiberboard; W = wollastonite; S = time intervals). Reprinted from [44] with permission of MDPI.

By evaluating the core composite temperature, they found a relevant difference among the medium-density fiberboards (MDF) panels and the wollastonite-treated panels (Figure 10A), due to higher thermal conductivity coefficient of this latter. Contrariwise, for the particleboard mats, they observed lower temperature with respect to the MDF ones (Figure 10B), attributing this behaviour to the higher contact surface among wood fibers (MDF matrix) compared with wood particles. In conclusion, they found a negative effect of 10%-feather content on physical and mechanical properties, whilst when the content of reinforcement was reduced to 5%, promising results were obtained which support that chicken feathers have potential in wood-composite panel production.

Rizal and co-workers designed and prepared biodegradable biopolymer films by using macroalgae. Since poor strength and moisture barrier of macroalgae, due to its hydrophilic nature, are well known they tried to overcome this problem by reinforcing films with natural fibers [45]. To this aim they employed polysaccharide microfibre (microcrystalline cellulose) derived from *Gigantochloa levis* bamboo (GL-MCC) and TGA thermograms evidenced, firstly, that that GL-MCC produced was free from lignin because of the absence of mass loss above 450 °C. Furthermore, by analyzing the thermogravimetric parameters, namely *T*_i_ and *T*_m_, they observed a comprehensive high thermal stability, compared to similar data reported in literature and attributed this good thermal performance to the high crystallinity of GL-MCC highlighted by X-RD. As regards the thermal stability of the plain-macroalgae reinforced with the GL-MCC, they observed a considerable increase of *T*_i_ and *T*_m_ that they justified with the strong intermolecular interactions between macroalgae matrix and GL-MCC. In the hypothesis of the authors the formed bonds increased the energy required to break the intermolecular bonding for the composite degradation, thus leading to an increase of the resistance to the thermal degradation. In conclusion, considering the high compatibility among macroalgae and GL-MCC and the increased rigidity of the macroalgae-based biopolymer films against microorganism and moisture attack, they proposed its use as a packaging material.

## 5. Thermal Techniques as Potential Tool for Biopolymers’ Design for Additive Manufacturing

In the last decades, the demand for petroleum resources replacement with eco-friendly materials has gone hand in hand with the rapid development in 3D printing technology, an additive manufacturing (AM) technique with a wide range of 3D structure fabrication and minimal waste generation [46,47].

**Figure 11:**
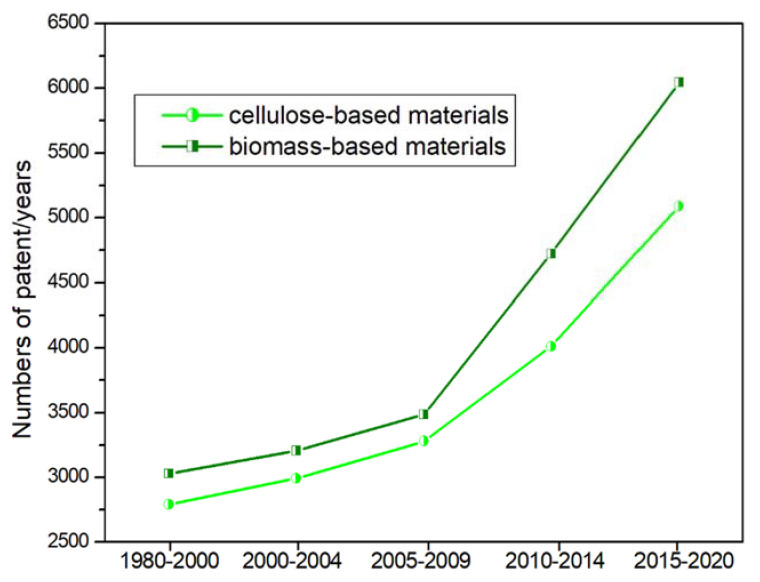
Time evolution of the patents deals with bio-based materials in AD.

Thus, both academic and industry sectors are widely employed in the development of bio-materials to be used in 3D printing technology as testified by the exponential increase of the patents deals with cellulose and biomass-derived materials for 3D printing (Figure 11). Simultaneous TG/DTA analyses were carried out by Cicala and co-workers to support the design of PLA based filaments for Fused deposition modeling (FDM) in comparison with commercial ones. TGA, in particular, was found to be an important tool to unveil filament’s composition. By analyzing the TGA traces, they observed an unusual, for PLA samples, residue at 600 °C thus assuming the presence of inorganic materials in the commercial formulation, that than they confirmed by FT-IR analysis. Always by using thermal techniques, namely DTA analyses, they confirmed the difference among the investigated compounds, in particular by evaluating *T*_g_, crystallization temperature and melting temperature they testified a good processability for two of the three investigated filaments, whilst the dramatic decrease in the melting in one of these filaments anticipated the worse printing behavior. They concluded that the thermal investigation proved determinative in the design and preparation of filaments to ensure a good printability [48].

**Figure 12:**
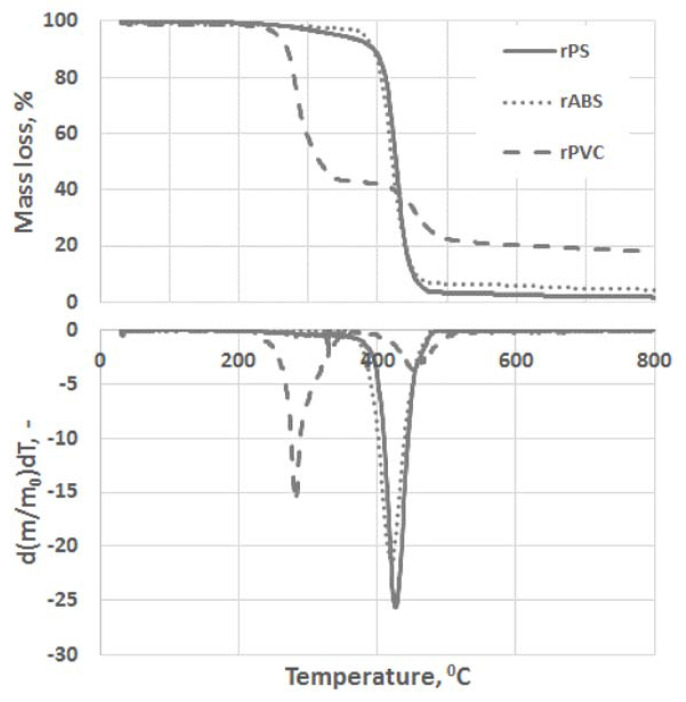
Thermogravimetric analysis (TGA) and differential thermogravimetry (DTG) curves of recycled Polystyrene (rPS), recycled acrylonitrile-butadiene-styrene (rABS) and recycled polyvinylchloride (rPVC) pyrolysis under a neutral atmosphere. Reprinted from [49] with permission of MDPI.

Turku et al. investigate the possible recyclability of vey common and used thermoplastics, such as polystyrene (PS), acrylonitrile butadiene styrene (ABS) and polyvinylchloride (PVC), for a possible re-use in 3D printing [49]. The chemical composition of the extruded filaments obtained by the recycled polymers was investigated by the means of DSC and TGA. The first technique allowed to the authors to establish the difference in temperature processability among the recycled and virgin materials, by evaluating *T*_g_. They observed a decrease in the *T*_g_ of the filaments derived from recycled PS and PVC than those of the respective control polymers, whilst *T*_g_ of recycled ABS filament was found to be similar with respect to that of control ABS. Then, by using TGA, the possible employment of PVC was definitively excluded due to the significantly faster mass loss rate compared to the ABS and PS grades (Figure 12).

They associated the worse thermal behavior of PVC to the low binding energy of the C–Cl responsible for the starting of dechlorination at low temperature and with the defects in the PVC structure formed during its polymerization (e.g., allylic and tertiary chloride moieties).

Since the microstructure of the fused filaments is largely affected by the cooling rates and the thermal gradients experienced across the part, de Jager et al. carried out a wide DSC investigation to evaluate the influences on the micro- and nano-structure of the crystallisation behaviour of Nylon-12 filaments [50]. DSC analysis allowed them to point out the limitations of non-isothermal crystallisation as a basis for fused filament fabrication (FFF), because of the range in printer chamber and the bed temperatures reached. Their studies evidenced significant differences in the shape of the melting endotherms for the non-isothermally crystallised samples and the FFF-produced ones. The authors hypothesized that these differences were due to the deposition-inducing effect, as well as the mesostructure cooling rates. In fact, during the printing process, the filament undergoes additional shear and therefore chain alignment, whilst it not affects the unit cell of the γ-form and may alter the crystallisation behaviour because regular chains are more likely to crystallise. Furthermore, they hypothesized that the reheating, caused by the continuous layer deposition, raise the filament temperature thus leading to complete crystallization. Once again, a thermal technique was determining to observe, for these authors, how a slow cooling rates lead to a better crystallite formation, evidencing also a divergence in thermal properties due to the range in printer chamber and bed temperatures attained during sample production.

## 6. Conclusions

Thermal techniques are widely used in the design, preparation, and characterization of polymeric materials. In this review, we highlight how in recent years the application field moved from petroleum-derived feedstock towards the bio ones. We reported the importance in determining thermal properties such as melting or decomposition temperature, crystallization temperature, activation energy of degradation and glass-transition temperature to proceed with a profitable biopolymers’ design, polymers’ recycling and preparation of recyclable polymers and as potential tool for biopolymers’ design in AM. The interest in this area is supported by a huge number of specific works and patents in which thermal techniques play a key role.

## Figures and Tables

**Table 1:** Popular techniques of thermal analysis.

Technique	Principle	Abbreviation
Differential Scanning Calorimetry	Heat flux difference	DSC
Differential Thermal Analysis	Temperature difference	DTA
Dynamic Mechanical Analysis	Oscillating force @ given T	DMA
Thermogravimetric Analysis	Mass loss	TGA
Thermal volatilisation analysis	Volatilisation rate	TVA

## Data Availability

Publicly available datasets were analyzed in this study. This data can be found in the cited references.

## References

[1] Blanco I. (2016). Lifetime prediction of food and beverage packaging wastes. J. Therm. Anal. Calorim..

[2] Blanco I. (2018). Lifetime Prediction of Polymers: To Bet, or Not to Bet—Is This the Question?. Materials.

[3] Pellis A., Malinconico M., Guarneri A., Gardossi L. (2021). Renewable polymers and plastics: Performance beyond the green. New Biotechnol..

[4] Falco G., Sbirrazzuoli N., Mija A. (2019). Biomass derived epoxy systems: From reactivity to final properties. Mater. Today Commun..

[5] Siracusa V., Blanco I. (2020). Bio-Polyethylene (Bio-PE), Bio-Polypropylene (Bio-PP) and Bio-Poly(ethylene terephthalate) (Bio-PET): Recent Developments in Bio-Based Polymers Analogous to Petroleum-Derived Ones for Packaging and Engineering Applications. Polymers.

[6] Finnveden G., Hauschild M.Z., Ekvall T., Guinee J., Heijungs R., Hellweg S., Koehler A., Pennington D., Suh S. (2009). Recent developments in Life Cycle Assessment. J. Environ. Manag..

[7] La Rosa A.D., Blanco I., Banatao D.R., Pastine S.J., Björklund A., Cicala G. (2018). Innovative Chemical Process for Recycling Thermosets Cured with Recyclamines^®^ by Converting Bio-Epoxy Composites in Reusable Thermoplastic—An LCA Study. Materials.

[8] Blanco I., Ingrao C., Siracusa V. (2020). Life-Cycle Assessment in the Polymeric Sector: A Comprehensive Review of Application Experiences on the Italian Scale. Polymers.

[9] Menczel J.D., Prime R.B. (2009). Thermal Analysis of Polymers. Fundamentals and Applications.

[10] Schlemmer D., Sales M.J.A. (2010). Thermoplastic starch films with vegetable oils of Brazilian Cerrado. J. Therm. Anal. Calorim..

[11] Cicala G., Saccullo G., Blanco I., Samal S., Battiato S., Dattilo S., Saake B. (2017). Polylactide/lignin blends. J. Therm. Anal. Calorim..

[12] Obradovic J., Voutilainen M., Virtanen P., Lassila L., Fardim P. (2017). Cellulose Fibre-Reinforced Biofoam for Structural Applications. Materials.

[13] Beber V.C., De Barros S., Banea M.D., Brede M., De Carvalho L.H., Hoffmann R., Costa A.R.M., Bezerra E.B., Silva I.D.S., Haag K. (2018). Effect of Babassu Natural Filler on PBAT/PHB Biodegradable Blends: An Investigation of Thermal, Mechanical, and Morphological Behavior. Materials.

[14] Amorim D.R.B., Bellucci F.S., Job A.E., da Silva Guimaraes I., da Cunha H.N. (2019). Electrical, structural and thermal properties of new conductive blends (PANICG) based on polyaniline and cashew gum for organic electronic. J. Therm. Anal. Calorim..

[15] Mothé C.G., de Freitas J.S. (2018). Lifetime prediction and kinetic parameters of thermal decomposition of cashew gum by thermal analysis. J. Therm. Anal. Calorim..

[16] Rafieian F., Mousavi M., Dufresne A., Yu Q. (2020). Polyethersulfone membrane embedded with amine functionalized microcrystalline cellulose. Int. J. Biol. Macromol..

[17] de Abreu Martins S., de La Caridad Om Tapanes N., Ribeiro Orlandini G. (2020). Study of the properties of an epoxy adhesive with additions of a residue from the biodiesel production process. Int. J. Adhes. Adhes..

[18] Bertolino V., Cavallaro G., Milioto S., Lazzara G. (2020). Polysaccharides/Halloysite nanotubes for smart bionanocomposite materials. Carbohydr. Polym..

[19] Zhao C., Huang C., Chen Q., Ingram I.D.V., Zeng X., Ren T., Xie H. (2020). Sustainable Aromatic Aliphatic Polyesters and Polyurethanes Prepared from Vanillin-Derived Diols via Green Catalysis. Polymers.

[20] Briones-Llorente R., Barbosa R., Almeida M., Montero García E.A., Rodríguez Saiz Á. (2020). Ecological Design of New Efficient Energy-Performance Construction Materials with Rigid Polyurethane Foam Waste. Polymers.

[21] Micó-Vicent B., Ramos M., Luzi F., Dominici F., Viqueira V., Torre L., Jiménez A., Puglia D., Garrigós M.C. (2020). Effect of Chlorophyll Hybrid Nanopigments from Broccoli Waste on Thermomechanical and Colour Behaviour of Polyester-Based Bionanocomposites. Polymers.

[22] Marzuki M.N.A., Tawakkal I.S.M.A., Basri M.S.M., Othman S.H., Kamarudin S.H., Lee C.H., Khalina A. (2020). The Effect of Jackfruit Skin Powder and Fiber Bleaching Treatment in PLA Composites with Incorporation of Thymol. Polymers.

[23] Soares A.P.S., de Freitas J.S., Mothé M.G., Mothe C.G. (2020). Thermal evaluation of composites from coffee capsules residue with sugarcane bagasse by TG/DTA and DMA. J. Therm. Anal. Calorim..

[24] Ignatyev I.A., Thielemans W., Vander Beke B. (2014). Recycling of Polymers: A Review. ChemSusChem.

[25] Day M., Cooney J.D., Fox J.L. (1994). The use of thermogravimetry to analyze a mixed plastic waste stream. J. Therm. Anal..

[26] Rostek E., Biernat K. (2013). Thermogravimetry as a Research Method in the Transformation Processes of Waste Rubber and Plastic Products for Energy Carriers (WtE and WtL Processes). J. Sustain. Dev. Energy Water Environ. Syst..

[27] Cafiero L., Castoldi E., Tuffi R., Vecchio Ciprioti S. (2014). Identification and characterization of plastics from small appliances and kinetic analysis of their thermally activated pyrolysis. Polym. Degrad. Stabil..

[28] Cafiero L., Fabbri D., Trinca E., Tuffi R., Ciprioti S.V. (2015). Thermal and spectroscopic (TG/DSC–FTIR) characterization of mixed plastics for materials and energy recovery under pyrolytic conditions. J. Therm. Anal. Calorim..

[29] Khaobang C., Areeprasert C. (2017). Investigation on thermal decomposition and kinetics study of recovered oil from electronic waste by thermogravimetric analysis. Energy Procedia.

[30] Rego A.S.C., de Santana A., Grillo A.V., Santosa B.F. (2019). Thermogravimetric Study of Raw and Recycled Polyethylene Using Genetic Algorithm for Kinetic Parameters Estimation. Chem. Eng. Trans..

[31] Kissinger H.E. (1957). Reaction kinetics in differential thermal analysis. Anal. Chem..

[32] Flynn J.H., Wall L.A. (1966). A quick direct method for the determination of activation energy from thermogravimetric data. J. Polym. Sci. B Polym. Lett..

[33] Ozawa T. (1965). A new method of analyzing thermogravimetric data. Bull. Chem. Soc. Jpn..

[34] Catauro M., Tranquillo E., Barrino F., Dal Poggetto G., Blanco I., Cicala G., Ognibene G., Recca G. (2019). Mechanical and thermal properties of fly ash-filled geopolymers. J. Therm. Anal. Calorim..

[35] Albitres G.A.V., Mendes L.C., Cestari S.P. (2017). Polymer blends of polyamide-6/Spandex fabric scraps and recycled poly(ethylene terephthalate). J. Therm. Anal. Calorim..

[36] Ma Z., Wang Y., Zhu J., Yu J., Hu Z. (2017). Bio-based epoxy vitrimers: Reprocessibility, controllable shape memory, and degradability. J. Polym. Sci. Part A Polym. Chem..

[37] Di Mauro C., Malburet S., Genua A., Graillot A., Mija A. (2020). Sustainable Series of New Epoxidized Vegetable Oil-Based Thermosets with Chemical Recycling Properties. Biomacromolecules.

[38] Di Mauro C., Tran T.-N., Graillot A., Mija A. (2020). Enhancing the Recyclability of a Vegetable Oil-Based Epoxy Thermoset through Initiator Influence. ACS Sustain. Chem. Eng..

[39] Lejeail M., Fischer H.R. (2021). Development of a completely recyclable glass fiber-reinforced epoxy thermoset composite. J. Appl. Polym. Sci..

[40] Souza D., Castillo T.E., Rodríguez R.J.S. (2012). Effects of hydroxyvalerate contents in thermal degradation kinetic of cellulose acetate propionate/poly(3-hydroxyalkanoates) blends. J. Therm. Anal. Calorim..

[41] Blanco I., Cicala G., Latteri A., El-Sabbagh A.M.M., Ziegmann G. (2017). Thermal characterization of a series of lignin-based polypropylene blends. J. Therm. Anal. Calorim..

[42] Cicala G., Tosto C., Latteri A., La Rosa A.D., Blanco I., Elsabbagh A., Russo P., Ziegmann G. (2017). Green Composites Based on Blends of Polypropylene with Liquid Wood Reinforced with Hemp Fibers: Thermomechanical Properties and the Effect of Recycling Cycles. Materials.

[43] Ge X., Chang M., Jiang W., Zhang B., Xing R., Bulin C. (2020). Investigation on two modification strategies for the reinforcement of biodegradable lignin/poly(lactic acid) blends. J. Appl. Polym. Sci..

[44] Taghiyari H.R., Majidi R., Esmailpour A., Samadi Y.S., Jahangiri A., Papadopoulos A.N. (2020). Engineering Composites Made from Wood and Chicken Feather Bonded with UF Resin Fortified with Wollastonite: A Novel Approach. Polymers.

[45] Rizal S., Lai T.K., Muksin U., Olaiya N., Abdullah C., Ikramullah, Yahya E.B., Chong E., Abdul Khalil H. (2020). Properties of Macroalgae Biopolymer Films Reinforcement with Polysaccharide Microfibre. Polymers.

[46] Blanco I., Cicala G., Ognibene G., Rapisarda M., Recca A. (2018). Thermal properties of polyetherimide/polycarbonate blends for advanced applications. Polym. Degrad. Stabil..

[47] Blanco I. (2020). The Use of Composite Materials in 3D Printing. J. Compos. Sci..

[48] Cicala G., Giordano D., Tosto C., Filippone G., Recca A., Blanco I. (2018). Polylactide (PLA) Filaments a Biobased Solution for Additive Manufacturing: Correlating Rheology and Thermomechanical Properties with Printing Quality. Materials.

[49] Turku I., Kasala S., Kärki T. (2018). Characterization of Polystyrene Wastes as Potential Extruded Feedstock Filament for 3D Printing. Recycling.

[50] de Jager B., Moxham T., Besnard C., Salvati E., Chen J., Dolbnya I.P., Korsunsky A.M. (2020). Synchrotron X-ray Scattering Analysis of Nylon-12 Crystallisation Variation Depending on 3D Printing Conditions. Polymers.

